# Evaluating the Efficacy of Certain Intermediate Irrigants in Preventing Precipitate Formed Due to Sodium Hypochlorite and Chlorhexidine Within the Root Canal System: An In Vitro Study

**DOI:** 10.7759/cureus.39871

**Published:** 2023-06-02

**Authors:** Rucha Patil, Sarita V Singh, Jyoti S Mandlik, Aniket Jadhav, Aishwarya Handa, Abhijeet Jadhav

**Affiliations:** 1 Department of Conservative Dentistry and Endodontics, Bharati Vidyapeeth Dental College and Hospital, Pune, IND

**Keywords:** stereomicroscope, edta, sodium thiosulfate, sodium hypochlorite, chlorhexidine

## Abstract

Aim:Root canal irrigation using a mixture of sodium hypochlorite (NaOCL) and chlorhexidine gluconate (CHX) might cause a precipitate to develop. This study aims to check the efficacy of sodium thiosulfate and normal saline as irrigating solutions.

Materials and methods: Roots of 45 teeth were biomechanically prepared, and the teeth were then tested. A size 15k file was introduced to the point where the apical foramen could be visible in order to provide an accurate reading of the working length. As a precaution against irrigating solutions leaking out, the specimens' tips were sealed with modeling wax before being instrumented. Each group's root canals were instrumented with #F4 hand Protaper (Dentsply Sirona, USA) as recommended by the manufacturer. Between instrumentation, The canals were lubricated with ethylenediamine tetraacetic acid (EDTA) and irrigated with 2.5% sodium hypochlorite (RC Help, Prime Dental, Mumbai, India). Fifteen samples were randomly assigned to one of three experimental categories based on the middle watering arrangement used: Group 1 (control), Group 2 (saline irrigant), and Group 3 (3.86% sodium thiosulfate). The jewel plate was submerged in water to cool it down, while two longitudinal scores were made on the root's buccal and lingual surfaces. We used a stereomicroscope to examine the exposed surfaces of the root trench in the coronal, middle, and apical thirds for the orange-earthy colored material (Stereozoom Nikon magnifying lens under 20X amplification), and we used the Mann-Whitney U test and the Kruskal Wallis test during our thorough analysis.

Results: The precipitation generated in the coronal, middle, and apical thirds had significantly different thicknesses. While precipitation did occur in all three regions, it was at a much lower rate in the apical third compared to the coronal and middle regions. In Group 1, the control group, the precipitate was thicker than in Groups 2 (saline irrigant) and 3 (3.86% sodium thiosulfate).

Conclusion: Sodium thiosulfate, which is a biocompatible solution, can be used as an intermediate irrigant as it shows less precipitate as compared to saline.

## Introduction

One of the primary goals of endodontic treatment is to disinfect the root canal system and remove any and all bacteria that may have been colonized there. Comprehensive canal disinfection warrants the use of irrigation along with mechanical cleaning to decrease the microbial load [1]. A biomechanical pretreatment is inadequate to remove resident bacteria and necrotic tissue from the root canal system due to the complexity of the system. This causes a layer of smear to form on the inside of the root canal [2]. Several irrigants have been used for canal disinfection over the years, one of which is sodium hypochlorite (NaOCl) at concentrations ranging from 0.5% to 6%, where it alone cannot cause so much of biofilm to be effectively removed [3,4]. Because of this, it is suggested that a solution that kills microbes, like sodium hypochlorite (NaOCl), be used along with biomechanical preparation to get rid of both the dead pulp and any germs that might be there [5,6].

Chlorhexidine gluconate (CHX), a cationic bisbiguanide, is an endodontic irrigant and an effective antimicrobial agent against resistant microorganisms, i.e. *Enterococcus faecalis* and *Candida albicans*, commonly found in infected root canals, were not poisonous; however, they could not break down organic material [7]. Therefore, it was suggested that NaOCl and CHX be used together to take advantage and get benefits from both irrigants. The interaction between NaOCl and CHX in the root canal system results in an orange-brown precipitated para-chloroaniline (PCA)-formulation, which is a by-product of the interaction. It causes cancer in humans and is damaging to the immune system [8,9]. The likelihood of coronal microleakage is raised because this precipitate may obstruct the dentinal tubules, creating a barrier between the filling material and the dentin surface. Tooth discoloration may come from PCA's presence in the root canal system, increasing the probability that unknown substances may leak into the peri-radicular tissue.

In order to eliminate this precipitate, certain intermediate irrigants, such as saline, citric acid, isopropyl alcohol, sodium metabisulfite, and sodium thiosulfate have been used. However, in this present study, saline and sodium thiosulfate have been used as intermediate irrigants between the interactions of NaOCl and CHX. The saline solution, even though it is widely used and advocated as an irrigant, has been seldom studied as an intermediate solution. Once upon a time, sodium thiosulfate was utilized as an antidote for cyanide poisoning and to alleviate calciphylaxis in patients undergoing hemodialysis for end-stage renal disease. Additionally, its neutralizing impact on NaOCl has been the subject of many laboratory experiments [10]. This research set out to assess the efficacy of sodium thiosulfate and saline solution in counteracting the precipitate generated by alternating applications of sodium hypochlorite and chlorhexidine gluconate (CHX) in the root canal system.

## Materials and methods

This study was carried out at Bharati Vidyapeeth Dental College and Hospital, Pune. Forty-five intact human permanent anterior teeth extracted due to periodontal reasons were selected. All 45 preserved specimens had their exteriors ultrasonically cleaned to remove any remaining tissue fragments, and then they were kept in triple-distilled water until the research was carried out. Teeth were ruled out if they had obvious flaws such as chips or fractures, abnormally shaped roots, numerous or split canals, an underdeveloped crown, or resorptive abnormalities.

To achieve an average root length of 10 mm or greater, at the point where cement and enamel meet, the teeth are adorned with a diamond disc. "The root canal walls were coronally flared with gates glidden drills #2 and #3. Using a #15k file, the canal was widened until it reached the apical foramen." The final working length was calculated by taking the total measured length and subtracting 1 mm. A gliding route was validated to a file size of at least 20k. Wax was used to seal the specimens' tips so that irrigating liquids wouldn't leak out and the samples could be handled with little effort. All of the root canals in the experimental groups were instrumented using hand Protaper instruments of size #F4 (Dentsply Sirona, USA). Before and after instrumentation, Using a hypodermic needle of 27 gauge, 2.5% sodium hypochlorite was injected into the canals, and ethylenediamine tetraacetic acid (EDTA) (RC Help, Prime Dental, Mumbai, India) was employed as the lubricant.

Using the following criteria, during the course of the experiment, 45 samples were divided evenly among three groups (n=15), with each receiving 2.5 ml of the intermediate irrigating solution and being left exposed to it for 60 seconds. Group 1 (control group): There was no intracanal irrigant utilized to intervene. Fifteen samples were irrigated with a solution containing 5.25% sodium hypochlorite and then 2% chlorhexidine gluconate. Group 2: Fifteen samples were irrigated first with 5.25% sodium hypochlorite, followed by an intermediary saline irrigant, and a final flush was done with 2% chlorhexidine gluconate. Group 3: Fifteen samples were irrigated first with 5.25% sodium hypochlorite, followed by an intermediary 3.86% sodium thiosulfate solution, and the final flush was done with 2% chlorhexidine gluconate.

**Figure 1:**
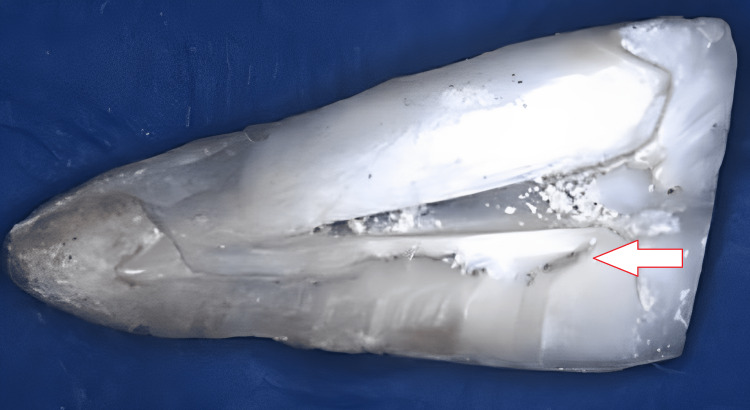
Stereomicroscopic imaging of the sample A group 3 (3.86% sodium thiosulfate) specimen showing no precipitation Arrow: dentinal and enamel junction

Preparation of 3.86% sodium thiosulfate: 3 grams of sodium thiosulfate crystals were added to 100 ml of distilled water, stirred for two minutes, and then stored in the amber-colored bottle. Each group's samples were dried with paper points, and then the imprint compound was employed to seal up the coronal opening. A diamond disc kept cold by water was used in this experiment. "There are two longitudinal grooves etched onto the root's buccal and lingual surfaces. The roots were divided with a hammer and chisel. The orange-brown precipitate was found by using a stereomicroscope to examine the root canal's exposed surfaces in the coronal, middle, and apical thirds (stereo zoom Nikon microscope under 20X magnification (Figure 1).

We made a series of uniaxial measurements from the outside surface of the precipitate to the interior dentinal wall. The photos were uploaded to a computer and edited using photo-editing software (MV Imaging-Stereo ver. 5.4.1) to determine how well they turned out.

Statistical analysis

In this investigation, both descriptive and inferential statistics were used. The data were analyzed using the Mann-Whitney U test and the Kruskal-Wallis test.

## Results

**Figure 2:**
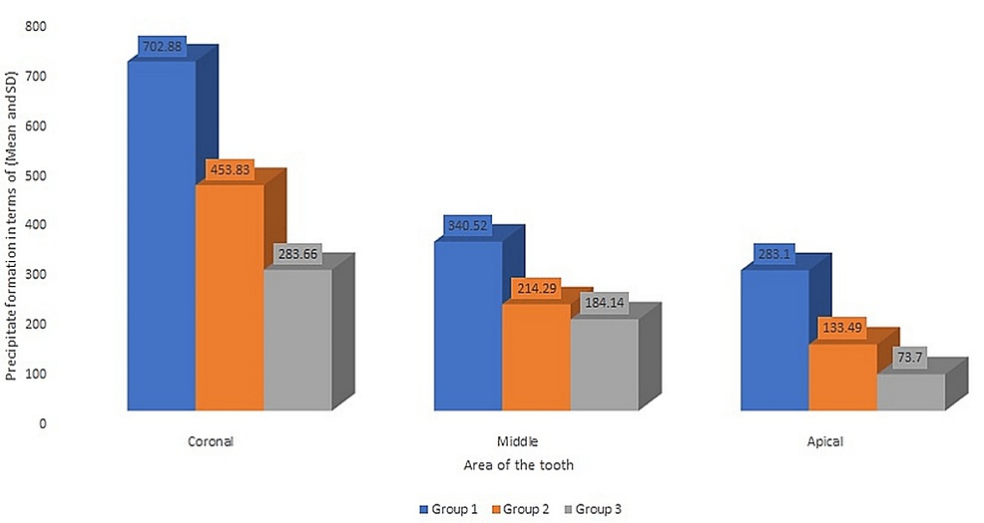
Comparison of the precipitate formation in terms of Mean (SD) at different levels in all the groups using Kruskal Wallis test

According to the results, Group 1 has the most precipitate formation compared to Group 2 and Group 3, while Group 3 has the lowest. The most precipitate was found in the coronal third of the root canal system across all three groups. We found that sodium thiosulfate worked better as an intermediate irrigant than regular salt water (Figure 2).

## Discussion

Challenges arise from the root canal system's complicated structure, which includes such features as isthmuses, fins, and curvatures [11]. An essential part of root canal therapy is chemo-mechanical cleansing and shaping [12]. The root canal system is a reservoir of a multitude of bacteria, bacterial contaminants, and necrotic debris and acts as a conduit for the spread of infection to other sites if not disinfected properly. The efficacy of several irrigants in the debridement and disinfection of canals has been studied. To find the best solution to utilize as an adjuvant to root canal therapy, researchers have analyzed each one's antibacterial and chemical activity and biocompatibility [13,14].

Single-rooted human permanent front teeth were chosen for this study because of their superior radicular mass and easier buccolingual root sectioning. In this section, we look for orange-brown precipitate (PCA) on the canal's radicular surface [15].

The instruments were used up to a size #F4 (0.40 mm) Protaper to let more irrigating fluid reach the apical third of the canal. Because it dissolves tissue in a unique way, sodium hypochlorite is a good irrigant for getting rid of dead tissue and biofilm. There is very little evidence that NaOCl at a concentration of 6% is superior to 5.25%. It has been noted that NaOCl solutions at concentrations of 0.5%, 3%, and 5% destroy the organic component (collagen) of dentine without mineral loss, potentially causing brittleness in teeth that have had endodontic treatment [14]. If NaOCl is expressed under pressure into the periodontal ligament gap, there are serious biological toxicity hazards when it is above 5.25 percent and it goes forcefully beyond the apex. With increasing focus, the results get noticeably worse [16-18]. However, when applied in high enough quantities, it may irritate the periapical tissues, it is not effective against certain germs when used at low doses, and it may corrode endodontic equipment [19]. CHX has been used as an endodontic irrigant because it kills a wide range of bacteria, has good lubricating and rheological properties, and is less toxic to cells than NaOCl. The concentration for CHX beyond 2% is not effective and more toxic in nature. CHX has been preferred over NaOCl, due to its biocompatibility, especially in cases of foramen enlargement, open apex, perforation, root resorption, or allergy when compared with other bleaching solutions. CHX is not highly bacteriocidal at 0.2%. Although CHX at a 2% concentration (chlorhexidine gluconate solution) is superior, it may produce a black precipitate that is difficult to remove if used immediately after NaOCl. When employing both NaOCl and CHX as irrigants in the same tooth, the authors advise using a saline-based intermediary. The inability of CHX to disintegrate organic tissue is a drawback. On the other hand, this contributes to the fact that it is of low toxicity to periapical tissues [20].

To maximize antimicrobial coverage, it has been suggested that endodontic treatment be performed using a mix of NaOCl and CHX as an irrigation regimen [21,22]. On the other hand, this combination produces a substantial precipitate. Numerous studies show that using 2% chlorhexidine as a final irrigating solution immediately after sodium hypochlorite causes the root canal surface to be coated and adversely impacts the permeability of the dentin. Possible negative effects on drug distribution and sealer performance [23,24]. Thus a need arose for an intermediary irrigant that would eliminate or reduce the precipitate formed and enhance the individual advantageous properties of both NaOCl and CHX as irrigants.

To avoid any chemical interactions between the two irrigating solutions, saline might be used as a buffer. Because it lacks tissue-dissolving or microbial-killing properties, it is unsuitable for use as a primary irrigant [25]. Saline and CHX irrigation alone form a precipitate due to the salting-out process. Group 2's flocculate thickness was lower than Group 1's in the present in vitro study, which is consistent with previous research by Mortenson et al. [26] and Khadse et al. [27] and can be explained by the use of saline as an intermediate irrigant, which dilutes the remaining sodium hypochlorite needed for the interaction with chlorhexidine.

Sodium thiosulfate as a medicament has been used in earlier clinical practices in the treatment of calciphylaxis [10,28]. It is unclear how sodium thiosulfate works, but two hypotheses have been put forth: either it increases calcium solubility in tissues, preventing its precipitation, or it creates a salt of thiosulfate of calcium (CaS_2_O_3_), which is very soluble and can be removed from the body via dialysis in people with end-stage renal disease [29,30]. When used as an intermediate irrigant, sodium thiosulfate decreased precipitation on canal walls compared to Groups 1 and 2. The removal of free chlorine ions as a result of the neutralizing action of sodium hypochlorite could be a probable explanation for this [31].

Furthermore, Krishnamurthy and Sudhakaran found results that were consistent with the present study, saying that the average thickness of the precipitate was greatest at the coronal and middle thirds and least at the apical levels [32]. This is explained by the fact that fewer irrigants reach the apex due to anatomical constraints and the constraints of the irrigation modality. Possible explanations include a lower concentration of CHX in the apical third due to its depletion in the coronal and middle thirds as a result of its interaction with NaOCl, and a higher concentration of NaOCl in the apical third [32]. Therefore, Both sodium thiosulfate and saline solution, when used as an intermediate endodontic irrigant in conjunction with chlorhexidine gluconate (CHX) and sodium hypochlorite (NaOCl), seem to have the ability to reduce the production of the orange-brown precipitate.

## Conclusions

Parachloroanaline, when formed, is carcinogenic and unfavorable and its formation can be prevented by the use of an intermediate neutralizing irrigant. Sodium thiosulfate (3.86%) proved to be a better intermediate irrigant when compared to saline. Within the limitations of this in-vitro study, it was observed that: i) The amount of precipitate formation is maximum in Group 1 (control group) than in Group 2 (NaOCl + Saline + CHX) and Group 3 (NaOCl + 3.86% sodium thiosulfate + CHX). ii) There is a statistically significant difference between the values of precipitate formation at the coronal and apical area of the root canal system in all the groups. iii) The P value is highly significant (< 0.001) at the coronal third, middle third, and apical third of the root canal system.

Hence, we conclude that: i) The interaction between sodium hypochlorite and chlorhexidine resulted in the formation of an orange-brown precipitate, that is difficult to remove and can compromise the seal of the obturated canal. ii) The formation of the precipitate can be prevented by the use of the saline solution and 3.86% sodium thiosulfate. iii) The use of saline minimizes the amount of precipitate formed by the interaction but cannot completely eliminate its presence. Sodium thiosulfate, which is a biocompatible solution, can be used as an intermediate irrigant as it shows less precipitate as compared to saline.
